# Radar-Based Fall Detection Using Micro-Doppler Signatures: A Comparative Analysis of YOLO Architectures

**DOI:** 10.3390/s26092650

**Published:** 2026-04-24

**Authors:** Ibrahim Seflek, Mücahid Barstuğan

**Affiliations:** Department of Electrical and Electronics Engineering, Faculty of Engineering and Natural Sciences, Konya Technical University, Konya 42250, Türkiye; iseflek@ktun.edu.tr

**Keywords:** elderly, fall detection, spectrogram, yolo, cw radar, human activity recognition

## Abstract

Human lifespan is increasing in parallel with the development levels of societies. Consequently, the number of elderly individuals worldwide is also rising day by day. One of the most significant risks these individuals face is falling. In this study, fall and daily activity data were collected from different home environments using a continuous-wave (CW) radar. Micro-Doppler signatures were generated from 700 data samples obtained from 10 individuals. Furthermore, the dataset was expanded by doubling the number of spectrogram images through data augmentation. The YOLO architecture, generally used in vision-based studies for object detection and tracking, was preferred for radar-based fall and activity detection. Classifications were performed with different YOLO structures, and comparative results are presented. At this stage, binary (fall/non-fall) and multi-class (seven different classes) classifications were carried out, achieving 100% accuracy for binary classification and 88.02% for multi-class classification. Additionally, the generalizability of the proposed architecture is demonstrated using the Leave-One-Subject-Out (LOSO) approach on the collected data and through the analysis of a public dataset. These results demonstrate the applicability of YOLO architectures in radar-based fall detection studies.

## 1. Introduction

Falls represent one of the most critical risks for the independently living elderly population, ranking as the second leading cause of unintentional injury deaths globally [1]. Given the rapidly growing elderly demographic [2], timely fall detection is crucial to minimize fatal outcomes and reduce the heavy burden of subsequent medical costs. Consequently, fall detection systems have gained significant research attention and are fundamentally categorized into wearable and contactless methods. Wearable approaches generally rely on sensors such as accelerometers, gyroscopes, and inertial measurement units (IMUs) to detect kinematic changes in the individual [3,4]. Often integrated into smartwatches or smartphones to ensure portability [5,6], these systems have proven to be effective. However, they possess inherent limitations, including inconsistent device placement, battery dependency, and the critical prerequisite that elderly users must consistently remember and be willing to wear them.

Contactless methods, conversely, utilize sensors or cameras to detect falls without requiring any user intervention. While microphones have been explored for this purpose [7,8], their high susceptibility to environmental noise limits their reliability compared to alternative sensors. Similarly, the utilization of LIDAR [9,10] presents challenges due to its high cost and operational complexity. Consequently, camera-based systems have become highly prominent in contactless fall detection [11,12]. In these optical systems, deep learning architectures, particularly convolutional neural networks (CNNs) and the You Only Look Once (YOLO) algorithm, are widely deployed. Several vision-based studies have proposed advanced architectures such as dual-stream networks, SAB-BiLSTM hybrids, and optimized YOLO variants that achieve exceptional recognition accuracy and mean Average Precision (mAP) in fall detection [13,14,15,16].

Despite their high classification success and apparent indispensability for emergency intervention, camera-based systems suffer from inherent limitations such as light sensitivity and vulnerability to visual occlusions. Furthermore, and perhaps most importantly, they inherently compromise user privacy, rendering them less viable for the continuous home monitoring of elderly individuals. This significant limitation necessitates the use of alternative, privacy-preserving contactless sensors. Consequently, radar technology has emerged as the most prominent contactless method in recent times, proving itself in numerous applications [17,18,19]. Compared to optical sensors, radar systems operate independently of ambient lighting, are robust against physical occlusions, and strictly preserve user privacy. Due to these distinct advantages, the theoretical framework and practical applications of portable microwave and millimeter-wave (mmWave) radars have rapidly expanded in contactless healthcare, successfully being utilized for sensing human micro-movements, such as gait smoothness [20].

### 1.1. Related Works

Radars, which have been employed in fall studies over the past nearly two decades, have yielded fruitful results during this period. A review of studies conducted in early fall reveals the utilization of a limited number of subjects, typically resulting in findings derived from a general machine learning approach founded upon data derived from a solitary environment. It is also observed that continuous-wave Doppler radars were utilized in majority of the studies. Liu et al. demonstrated the presence of Doppler signatures by employing Mel-frequency cepstral coefficients as a means of analysis. The performance of fall detection was facilitated by the utilization of Support Vector Machine (SVM) and K-Nearest Neighbor (KNN) as classifiers [21]. Gadde et al. presented a paper on the use of Mahalanobis distance and wavelet transform for the purpose of fall detection [22]. Another study on high-accuracy fall detection presented spectrograms classified with discrete prolate spherical sequences using an SVM classifier. Spectrograms are obtained using a multi-window approach based on Slepian and Hermite functions. Furthermore, spectrograms are favored for fall detection by reconstructing absent samples using the multiple measurement vector (MMV) model [23].

Recent studies have seen an increase in the number of subjects and measurement environments. In addition to the use of continuous-wave radars, the introduction of commercial off-the-shelf radars has demonstrated the impact of frequency-modulated continuous-wave (FMCW) and ultra-wideband radars with MIMO structures on research. Furthermore, deep learning methods, which eliminate the need for manual feature extraction, have made a significant mark on the literature. Fall detection studies have gained momentum and achieved considerable success by utilizing point cloud approaches with CNN, RNN, and similar methods, in addition to spectrograms and maps such as Doppler–time, Doppler–range, and range–angle obtained from raw data. In their study, Jokanovic et al. presented various architectures employing spectrograms and range maps in the context of deep learning. The study incorporates a two-layer auto encoder and logistic regression. The study utilized three subjects, four activities, and 408 data, and it was demonstrated that fall detection achieved a maximum success rate of 97.1% in various scenarios [24]. Sadrezami et al. developed a fall detection system that utilizes ultra-wideband (UWB) radar and convolutional neural networks (CNNs). In their approach, spectrograms are converted into binary images, thereby facilitating the identification of fall-related events. The researchers proposed a novel methodology that distinguished falls from non-falls with 98.37% sensitivity and 97.82% specificity. The study concluded that the proposed method yielded superior results in comparison to existing methodologies [25]. In another study using multiple UWB radars in a home environment, fall detection is performed using the CNN-LSTM method, combining the power of CNN and LSTM. The study presents three different strategies and achieves an accuracy rate of approximately 90% [26].

Kittiyanpunya et al. conducted a series of experiments on ten subjects across five different rooms, utilizing an FMCW radar to demonstrate fall detection by employing the point cloud approach. The classification of falls and other activities is facilitated by 1-D point clouds and Doppler velocity, with the assistance of LSTM. It was documented that their proposed technique attained a fall detection accuracy of 99.50% [27]. In a study aiming to both track humans and detect falls, a dynamic Density-Based Spatial Clustering of Applications with Noise (DBSCAN) based on signal SNR levels is proposed using multiple FMCW radars. Scenarios such as target state prediction for fall detection and a feedback loop for noise reduction are presented. The study presents fall detection with 96% accuracy. Fall position estimation using 3D point clouds is also demonstrated [28].

In the study by Li et al., spectrograms are derived from raw radar data, with CNN and BiLSTM network architectures employed for the extraction of spatial features and temporal sequential features, respectively. This approach, designated as CB-LSTM, substantiates the feasibility of concurrently utilizing both temporal sequential and spatial features. It has been asserted that the proposed method attains a 98.83% level of accuracy and occupies a significant position within the extant literature on the subject [29]. In a further study using spectrograms, CW radar is used to perform binary classification of five fall events and six non-fall events. The study uses a lightweight fall detection method based on a binary neural network (BNN) and achieved 93.1% accuracy. It is also claimed that the study was performed with lower memory usage [30]. Zhao et al. present mmFall for fall detection. The method uses range–angle (RA) energy maps to separate multiple moving targets, determine their locations, and predict their states more accurately. In the study where YOLO is used for human detection, falls are detected using a deep neural network (DNN) architecture. The method, presented with an extensive measurement study, offers fall detection with an average recall of 0.969 and a precision of 0.996 [31].

### 1.2. Motivation of the Study

A review of the literature on fall detection reveals that studies employing deep learning algorithms are of particular note. In radar-based studies, spectrograms and data maps, alongside architectures like traditional CNNs and LSTMs, are used extensively. In the domain of camera applications, the YOLO architecture is renowned for its exceptional speed and optimized computational load in object detection. However, the implementation of YOLO in radar studies remains remarkably minimal.

The core motivation of this study is not merely to apply an unexplored algorithm but rather to exploit the distinct architectural advantages of YOLO for radar signal processing. Traditional radar fall detection often requires complex, heuristic signal processing pipelines to extract temporal and spatial features. By converting 1D continuous-wave radar data into 2D spectrogram images, the complex signal processing load is bypassed and shifted to a highly optimized image classification pipeline. YOLO’s grid-based feature extraction offers a lower computational burden and faster inference times compared to multi-stage CNN-LSTM hybrids, making it exceptionally suited for real-time, low-resource continuous monitoring.

To the best of our knowledge, this is the first study where CW radar data is converted into spectrograms, augmented via thresholding, and directly classified using YOLO models for fall and activity detection. The present study employs a low-cost CW radar to obtain a total of 700 fall and daily activity data samples through measurements in two different residential environments. Spectrograms are derived from the obtained data. Furthermore, thresholding is performed on the spectrograms for data augmentation. The detection of falls and daily activities is presented comparatively using multiple YOLO variants with classification capabilities. The contributions of this study can be summarized as follows:

- Experimentally validating the elevated success rate of YOLO architectures, which are conventionally employed for optical-based object detection and tracking, when adapted as a classifier on radar data (e.g., spectrograms) for fall detection and activity classification.
- Proposing a thresholding-based data augmentation method to expand the limited radar dataset and to analyze the consistency and performance of the model trained with the augmented data compared to the model trained with the original dataset.
- Demonstrating the ability to perform high-accuracy fall detection and activity classification using a cost-effective and accessible CW radar sensor, compared to complex radar systems.
- Verifying the robustness of the proposed method against environmental differences and movement patterns that vary from person to person, and confirming the generalizability of the system by testing it in different environments and on multiple subjects.
- Establishing the extensive generalizability and reliability of the proposed architecture by implementing the Leave-One-Subject-Out (LOSO) cross-validation approach on the collected data and further verifying the model’s performance on an independent public dataset.

The paper is organized as follows: The features of radar environments and data collection are presented in Section 2. The classification method and creation of spectrogram images are explained in Section 3. The experimental study and results are discussed in Section 4. The concluding remarks and the literature comparison are presented in Section 5.

## 2. Experimental Setup and Measurement

### 2.1. Continuous-Wave (CW) Radar

**Figure 1:**
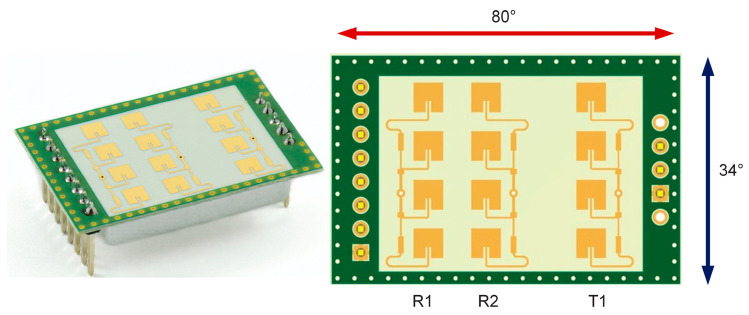
RFbeam K-LD7 radar [32].

The K-LD7 radar, a commercial off-the-shelf product from RFbeam Microwave, is employed in the collection of falls and daily activity data. Operating within the 24 GHz band, the radar is distinguished by its low power consumption and is equipped with a 3 × 4 patch antenna configuration. In addition, the antenna’s asymmetric structure, with a horizontal beam width of 80° and a vertical beam width of 34°, ensures a comprehensive detection area. The system comprises one transmitter and two receivers. This allows the radar, which uses Frequency Step Modulation (FSK) for distance measurement, to provide a detection range of up to 15 m for people. In this study, data is collected in CW mode using one transmitter and one receiver of the radar. The radar in question features a quadrature receiver structure, yielding I (in-phase) and Q (quadrature) outputs with a 90° phase difference between them. Consequently, this configuration is often the preferred option for a wide range of radar applications [32]. Figure 1 illustrates the radar unit employed for fall and activity detection in this study.

### 2.2. Measurement Environments

**Figure 2:**
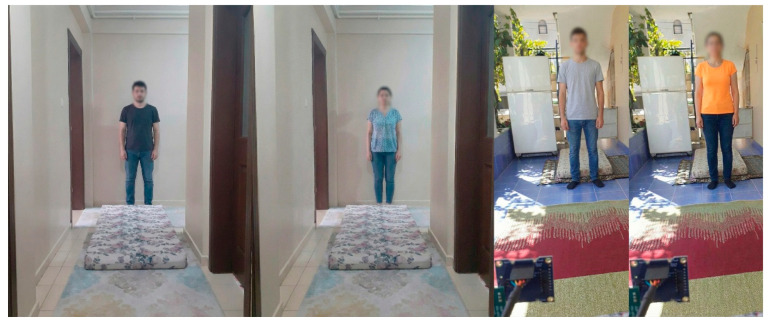
Measurement environments.

The measurement process is conducted in two distinct environments to evaluate fall detection performance. Conducting measurements in diverse settings demonstrates the generalizability of the study. Furthermore, a home environment is considered more realistic than a laboratory environment for conducting such experiments. The environments are selected to effectively represent falls and daily activities. The first environment is the entrance hall of an apartment with dimensions of 1.7 m × 5 m. The second environment is the balcony area at the entrance of a detached house, measuring 2.8 m × 5 m. To ensure consistency throughout the measurement process, the same measurement parameters are employed in both environments. Figure 2 displays the measurement environments.

### 2.3. Data Collection

The collection of fall and daily activity data was conducted in two distinct environments, with ten healthy individuals participating in the study. Six subjects participated in the first environment, and four subjects participated in the second environment. The subjects are healthy men and women aged 18–38. The subjects’ profiles are delineated in Table 1.

The prevalence of young and healthy individuals simulating falls and daily activities is a standard practice in the literature [23,24,25,26,27,28,29,30,31]. Although research is being conducted on detecting falls in the elderly, subjecting these individuals to such a risky situation is ethically and medically nearly impossible, considering that this situation is difficult and risky even for young people. Consequently, research in the extant literature continues with this standard practice. In this study, the four most common falls (forward, backward, right, and left) and three non-fall movements (walking, sitting–standing, and crawling) encountered in the literature were performed by the subjects. Each subject is requested to repeat the movements ten times. Consequently, a total of 700 falling and non-falling movements were obtained in the study.

**Figure 3:**
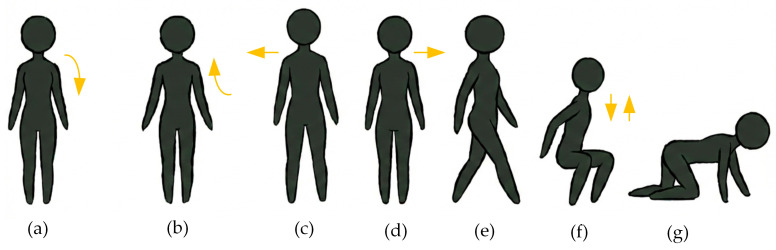
Falls and other daily activities: (**a**) falling forward, (**b**) falling backward, (**c**) falling to the right, (**d**) falling to the left, (**e**) walking, (**f**) sit and stand, (**g**) crawling.

Each class initially contained 100 samples. After the data augmentation process, the number of samples in each class increased to 200. At the multi-class classification stage, the number of samples in each class is equal. At the binary classification stage, the dataset was prepared with 400 samples for the fall class and 300 samples for the non-fall class. Therefore, there was no class imbalance in the dataset. Similarly, after the data augmentation process, 800 samples were obtained for the fall class and 600 samples for the non-fall class. The movements accomplished in the study are depicted in Figure 3.

The measurements were taken in succession; subjects were requested to move on to the next movement after completing each repetition. It is observed that the movements are quite challenging and strenuous, even for young individuals.

The radar is positioned 90 cm above the ground, with each subject directly in the radar’s field of view. In the context of experimental procedures involving falls, a cushion is used to ensure the safety of the subject and to prevent potential injury. Fall and sitting–standing measurements are performed at a distance of three meters from the radar. This distance is regarded as being suitable for a domestic environment. The distance is set at 2–4 m to allow for comfortable performance of other activities. Each measurement is taken at a rate of 11.5 s and is sampled at a frequency of 2200 Hz. The measurement and data collection strategy is performed in the same way in both environments.

## 3. Proposed Method

**Figure 4:**
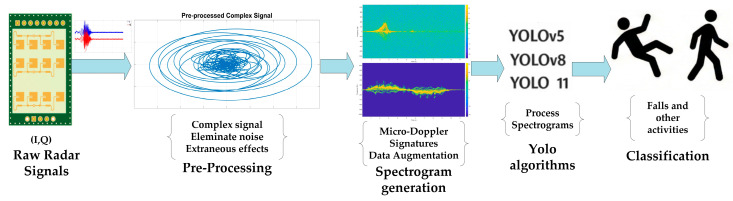
Block diagram of the proposed method.

The initial stage of the proposed method process involves the preprocessing of raw radar data, with the objective of eliminating noise and other extraneous effects. Spectrograms are then obtained to generate micro-Doppler signatures resulting from human falls and other activities. The YOLO algorithms are then utilized to process the spectrograms, with the objective of detecting and classifying falls and other activities. The proposed method’s block diagram is presented in Figure 4.

### 3.1. Preprocessing

The raw *I* and *Q* signals obtained from falls and other activities are combined to form a complex signal prior to further processing.

(1) C(n)=I+jQ

In order to eliminate undesirable components and potential misidentifications caused by DC components from the resulting complex signal, the signals are averaged and then subtracted from the existing signals.

(2) C(n)=C(n)−mean(C(n))

Subsequently, a moving average filter is applied to the signal from which the DC component is removed, in order to remove noise and make the signal more understandable and smoother.

(3) $$C\left[n\right]=\frac{1}{L}\sum_{m=-M}^{M}x\left[n+m\right]$$

In this instance, the filter length is defined as *L* = 2*M* + 1. The method is predicated on the principle of replacing each point on the signal with the average of that point and a specified number of its neighbors.

### 3.2. Spectrogram

**Figure 5:**
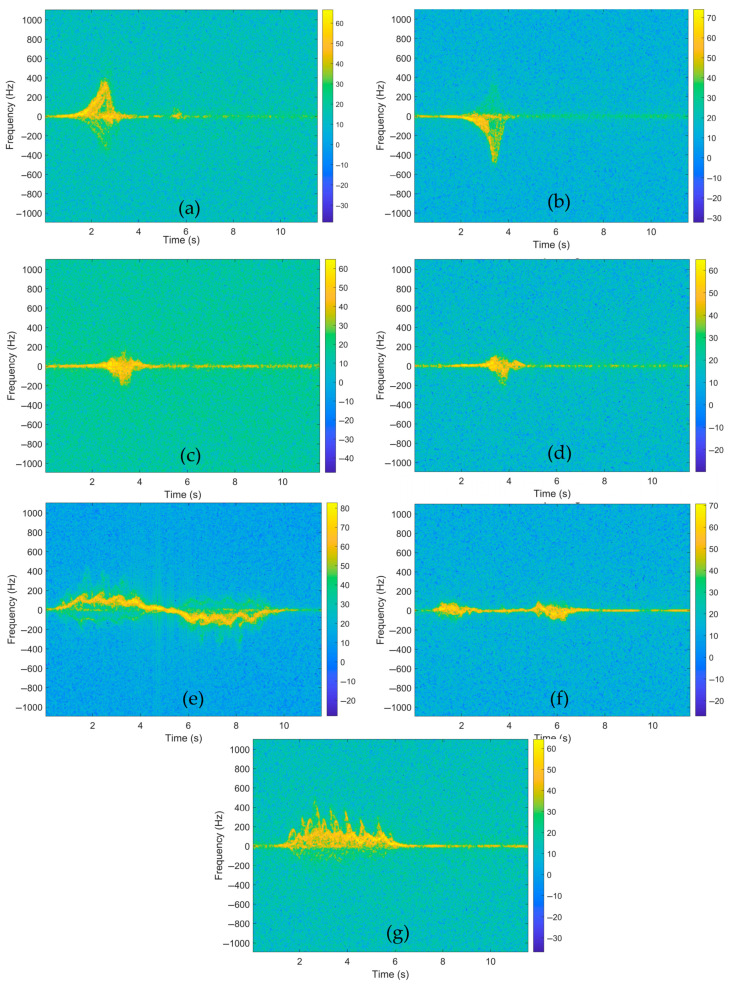
Spectrograms for falls and other daily activities: (**a**) falling forward, (**b**) falling backward, (**c**) falling to the right, (**d**) falling to the left, (**e**) walking, (**f**) sit and stand, (**g**) crawling.

In the realm of radar-based fall and daily activity detection, the analysis of time-dependent Doppler frequency variations caused by human movement assumes paramount importance. In this context, micro-Doppler signatures are revealed through methods based on time–frequency analysis. Spectrograms are considered to be one of the most prevalent and favored forms of time–frequency representation. A spectrogram is a two-dimensional image derived from the square of the magnitude of the Short-Time Fourier Transform (STFT), which visualizes the power density of a radar backscatter signal as a function of both time and frequency. The spectrogram of a signal *C*(*n*), *n* = 0… *N* − 1, is represented as follows:

(4) $$Spc\left(n,l\right)=\;{\left|\sum_{r=0}^{N-1}w\left(r\right)C\left(n-r\right)e^{-j2\pi lr/N}\right|}^{2}$$

where *w*(*r*) is a window function. Spectrograms are regarded as a fundamental component in radar-based fall detection systems, both in terms of physical interpretability and classification performance. The parameters employed in the study for spectrogram construction are outlined in Table 2. The spectrograms obtained for falls and daily activities are displayed in Figure 5.

### 3.3. Data Augmentation

The efficacy of deep learning models is contingent upon the availability of substantial amounts of labeled data, which is essential for the model to make effective generalizations. However, in radar-based fall detection scenarios, this requirement is often difficult to meet due to the challenge of obtaining large-scale experimental datasets. Standard data enhancement techniques, such as geometric transformations (e.g., rotation or flipping), frequently used in computer vision, are not always physically meaningful for micro-Doppler signatures because they have the potential to disrupt the kinematic integrity of the fall motion. Therefore, to expand the training dataset and improve the model’s resilience to environmental clutter, a signal processing-based data enhancement strategy focused on Spectrogram Thresholding is implemented. This approach effectively increases the size of the dataset by creating a secondary, fairly low-noise view of each example.

As delineated in (4), the term *Spc*(*n*,*l*) is employed to denote the radar signal’s initial spectrogram. In order to facilitate the visualization of the high dynamic range variations in radar signals, the logarithmic power spectrum, *PdB*(*n*,*l*), is calculated as follows:

(5) $$P_{dB}(n,l)=10\log_{10}(Spc(n,l))$$

In order to isolate the Region of Interest (ROI) containing the micro-Doppler signature from background noise and implement the data augmentation process, a binary masking function based on a predefined power threshold (*γ*) is defined. The resulting augmented spectrogram, *Paug*(*n*,*l*), is expressed as follows:

(6) $$P_{aug}(n,l)=\left\{\begin{array}{ll} P_{dB}(n,l),\;\;\;\;\;\; & if\;P_{dB}(n,l)\geq \gamma \\ \eta ,\;\;\;\;\;\;\;\;\; & if\;P_{dB}(n,l)<\gamma \end{array}\right\}$$

In our experimental setup, based on the environmental noise floor, the threshold value *γ* is empirically determined to be 32 dB. The augmented spectrograms are generated by preserving the original high-energy components while suppressing sub-threshold regions to a fixed noise floor value (*η* = −45 dB). By appending these thresholded samples to the original raw dataset, the final training set contains pairs of both noisy and clean representations for each event. Preliminary experiments indicate that threshold values below 32 dB are insufficient to effectively suppress noise and clutter in the spectrograms, resulting in reduced signal quality. Conversely, threshold values above this level tend to remove weak but important micro-Doppler components associated with limb movements, negatively affecting the discriminative information in the data.

**Figure 6:**
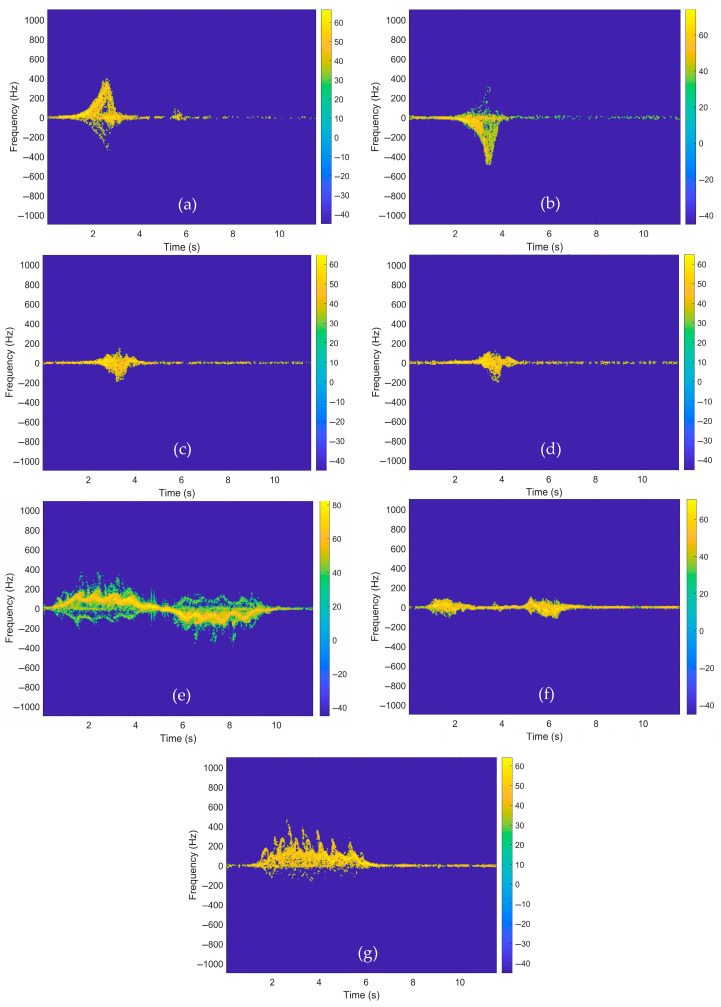
Thresholded spectrograms: (**a**) falling forward, (**b**) falling backward, (**c**) falling to the right, (**d**) falling to the left, (**e**) walking, (**f**) sit and stand, (**g**) crawling.

This strategy fulfills two functions: The first is that it reduces the risk of overfitting by increasing sample diversity. The second is that it forces the neural network to learn features that are invariant to background noise levels, focusing on the morphological structure of the micro-Doppler signature rather than environmental artifacts. The spectrograms obtained through the implementation of thresholding are presented in Figure 6.

### 3.4. You Only Look Once (YOLO)

The You Only Look Once (YOLO) method is a single-stage deep learning framework first proposed for real-time object detection, and later developed for classification tasks. YOLO processes the input image in one step, unlike two-step approaches. By doing so, it learns feature extraction and classification stages simultaneously, which provides high speed image processing [33].

YOLO includes three main components: backbone, neck, and head. Backbone extracts hierarchical feature representations on the input image by using convolutional neural networks (CNNs). The neck aggregates multi-scale features by using feature pyramid structures to enhance robustness against scale variations. Finally, the head performs localization, confidence score calculation, and classification [33]. This modular structure provides YOLO models that learn discriminative features while maintaining computational efficiency, making them implementable for real-time and offline object detection, segmentation, and classification tasks.

This study used YOLOv5, YOLOv8, and YOLO11 classifier structures to obtain high classification accuracy on spectrogram images. There are differences between these three YOLO models.

**Figure 7:**
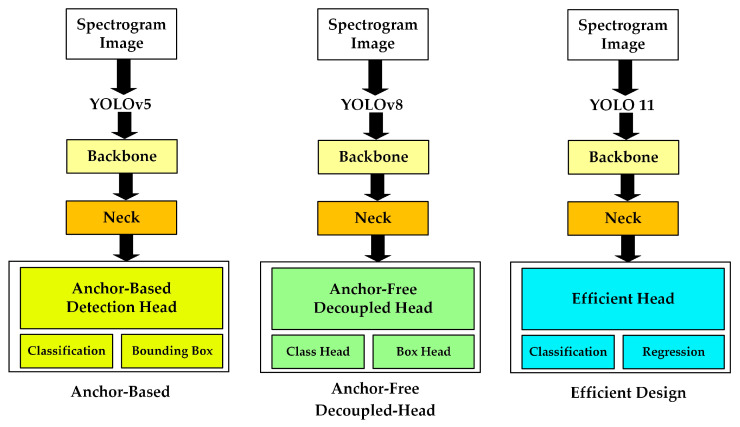
The summarized YOLO model architectures.

YOLOv5 employs an anchor-based detection head and CSP-based backbone structures. It offers stable training behavior and competitive performance, and its reliance on predefined anchor boxes introduces additional hyperparameter tuning and may limit adaptability across datasets. YOLOv8 provides several architectural improvements compared to the previous versions. The most effective part is that it adopts an anchor-free and decoupled head design, separating classification and regression tasks into independent branches. This reduces dataset dependency, increases training performance, and provides more accurate feature representation. YOLO11 focuses on further architectural optimization and computational efficiency. It provides reduced model complexity and improved inference performance. Figure 7 shows the summarized YOLO model architectures used in this study.

All YOLO models were trained using identical datasets, preprocessing steps, and training parameters to ensure a fair comparison. Pretrained weights were utilized to accelerate convergence and stabilize training. Model performance was evaluated using standard classification metrics, including accuracy, precision, recall, and inference time.

## 4. Experimental Results and Discussion

In this study, three different datasets containing original and filtered (thresholded) spectrogram images were created. Each dataset was divided into two separate sub-classification sets: two-class and seven-class sets. The first dataset contained the original spectrogram images, the second contained the filtered spectrogram images, and the third contained both original and filtered spectrogram images. Three different datasets, each with two separate sub-classification sets, have a total of six different classification sets. The created datasets are classified using different YOLOv5-cls, YOLOv8-cls, and YOLO11-cls models with five-fold cross-validation.

Table 3 presents the number of training, validation, and test images during both multi-class and binary classification stages.

### 4.1. Evaluation Metrics

The performance of classification is calculated using a confusion matrix that provides predicted and ground truth class labels. The predictions are categorized into true positives (TPs), true negatives (TNs), false positives (FPs), and false negatives (FNs) for a given class. A true positive represents a correctly classified sample belonging to the target class, while a false positive refers to the incorrect assignment of a sample belonging to a different class within the target class. False negatives indicate missed samples belonging to the target class, while true negatives represent non-target samples that were correctly rejected.

The overall classification performance is calculated using the accuracy proportion of correctly classified samples over the total number of samples in the dataset. The accuracy metric is calculated as follows:

(7) $$Accuracy=\frac{\mathrm{TP}+\mathrm{TN}}{\mathrm{TP}+\mathrm{FP}+\mathrm{FN}+\mathrm{TN}}$$

Although accuracy provides an intuitive performance indicator, it may be misleading in the presence of class imbalance. Therefore, additional metrics (precision and recall) are required for a more reliable evaluation. Precision quantifies the reliability of positive predictions by measuring the proportion of correctly predicted samples among all samples predicted as a given class. Precision is defined as

(8) $$Precision=\frac{\mathrm{TP}}{\mathrm{TP}+\mathrm{FP}}$$

High precision indicates that the model produces a low number of false positives, which is particularly important in applications where incorrect class assignments are costly. Recall, also referred to as sensitivity, measures the model’s ability to correctly identify all samples belonging to a given class. Recall is defined as

(9) $$Recall=\frac{\mathrm{TP}}{\mathrm{TP}+\mathrm{FN}}$$

A high recall value indicates that the model successfully captures most instances of the target class, reducing the number of missed classifications.

In multi-class classifications, each metric can be calculated separately and, subsequently, aggregated for weighted-averaging or macro-averaging strategies. Macro-averaging metrics evaluate all classes equally, while weighted averaging considers class frequencies, which provides a more representative measure in imbalanced datasets.

### 4.2. The Structural Parameters for YOLO Models

Table 4 compares the architectural and computational features of YOLOv5n-cls, YOLOv8n-cls, and YOLO11n-cls models based on the classification task. Models have a similar number of parameters. However, architectural design is different. YOLOv8-cls provides a more stable and efficient training process thanks to its simplified backbone design and balanced parameter computation cost. This situation aligns with the experimental results, showing that the YOLOv8-cls model achieved the highest classification performance. Although YOLO11-cls is computationally advantageous with its lower FLOP value, it could not reach the level of YOLOv8-cls under the dataset and training configuration used in this study. YOLOv5-cls, being based on an older architecture, lagged behind in terms of classification performance.

Each YOLO architecture was trained with pretrained weights. The initial learning rate was set to 0.01, and a cosine decay schedule was applied throughout the training process. Optimization was performed using Stochastic Gradient Descent (SGD) with momentum. Due to hardware limitations, the batch size was set to 4. The training process was conducted for a maximum of 100 epochs, with early stopping employed using a patience value of 20 epochs to prevent overfitting.

### 4.3. Analysis of Training and Test Processes

In the classification phase, the seven-class dataset consisting of original spectrogram images is first classified using all sub-models (nano, small, medium, large, xlarge) of YOLOv8 with five-fold cross-validation. The validation and test results obtained from the five-fold cross-validation are presented in Table 5.

As shown in Table 5, the best classification result was obtained with the nano model in both the validation and testing phases. Therefore, the nano model of each YOLO architecture was used in the remaining five different classification sets.

The validation and testing results obtained from the classification process using the YOLOv5n, YOLOv8n, and YOLO11n models for the other classification sets are presented in Table 6 and Table 7.

Table 6 presents the validation results obtained using the five-fold cross-validation method. The table compares the performance of the YOLOv5n-cls, YOLOv8n-cls, and YOLO11n-cls models in both multi-class and binary classification tasks.

When the multi-class classification results are examined, it is observed that all models exhibit successful performance with accuracy rates ranging from 88% to 93%. The YOLOv8n-cls model achieves the highest performance with an average accuracy rate of 92.86% on the original dataset. This result demonstrates that the improved feature extraction capabilities of the YOLOv8 architecture provide high generalization success on the validation set. The YOLO11n-cls model also exhibits similar success with an average accuracy of 92.02%, thereby confirming that next-generation YOLO architectures offer strong performance for multi-class classification problems.

**Figure 8:**
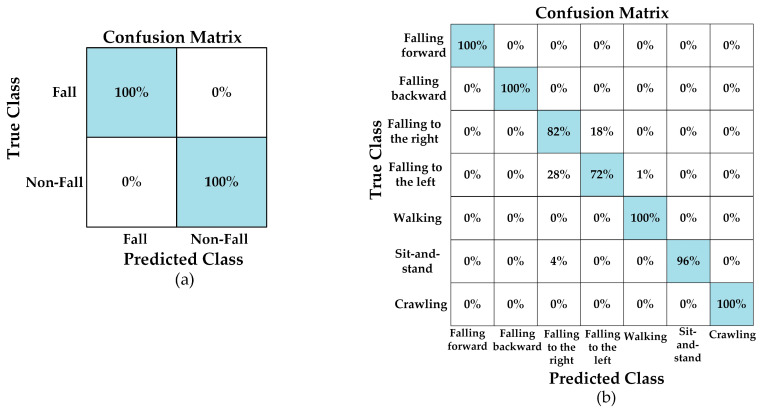
The confusion matrix results of (**a**) binary (YOLOv5, YOLO11) and (**b**) multi-class (YOLOv8) classifications for original fall data during validation process.

An overall increase in the stability of the accuracy values of the models is observed after the filtering process. In particular, the fact that the YOLO11n model has achieved an accuracy rate of 92.28% on filtered data shows that the data preprocessing (filtering) step significantly contributes to the model’s performance. This increase suggests that the filtering process reduces noise, allowing for clearer learning of the distinguishing features between classes. On the other hand, the slight decrease in accuracy after filtering in the YOLOv8n model (89.9%) indicates that the model is better adapted to the diversity of the original data. In the “Original + Filtered” dataset, the YOLOv5n-cls model showed a more stable performance compared to other models with an average accuracy rate of 91.58%. This shows that the YOLOv5 architecture can produce stable results even in mixed (heterogeneous) datasets. In addition, the five-fold validation results of all models are quite close to each other, indicating that the models show high stability and repeatability against random data splitting. For the binary classification task, all models exhibited almost flawless success. Accuracy rates ranging from 98.6% to 100% are achieved across all data types. In particular, the YOLO11n-cls model achieved the highest performance with 100% accuracy across all classes, learning the difference between two classes almost perfectly. This result demonstrates that the models strongly capture class differentiation on the dataset and that the system has high reliability in practical use. Figure 8 shows the confusion matrix for each classification result after the validation process.

Figure 8 shows that there is high-accuracy performance for five classes, where the model gives balanced results during multi-class classification. The classification method accurately separated classes of “falling forward”, “falling backward”, “walking”, “sit-and-stand”, and “crawling”. The pattern of these activities has more discriminative features than the others. However, there is a performance decrease for classification of “falling to the right” and “falling to the left” classes. The method detects the fall but cannot classify the direction with high accuracy. This predicament arises due to the inherent limitations of CW radar in acquiring range and location information; it is only capable of measuring radial velocity. This complicates the distinction between right and left falls. The balance between precision (93%) and recall (92.8%) shows that the model does not miss the fall. However, it cannot catch the direction of the fall, whether it occurs on the right or left side.

In conclusion, the validation results show that the YOLOv8n and YOLO11n models can achieve high-accuracy rates for multi-class classification tasks, that the filtering process particularly improves model stability, and that all models have excellent generalization capabilities in binary classification. These findings indicate that the developed classification system is optimized in terms of both data quality and model architecture and has a strong performance foundation for real-world applications. Table 7 shows the results of the five-fold cross-validation performed on the test set and presents a comparative performance of the YOLOv5n-cls, YOLOv8n-cls, and YOLO11n-cls models for both multi-class and binary classification tasks.

Table 7 shows that for the multi-class classification task, the accuracy rates of the models generally range from 43% to 88%. The highest average accuracy rate is obtained with the YOLOv8n-cls model (88.02%), followed by the YOLO11n-cls model (86.14%). This result demonstrates that the YOLOv8n architecture is superior to other models in terms of generalization ability on the test data. It was observed that general stability in accuracy rates was achieved, and the stability of the models increased when filtering was applied. In particular, the fact that the YOLOv8n-cls model achieved an accuracy of 87.58% on the filtered data reveals that this model can consistently perform at a high level with noise-free data. However, the YOLO11n-cls model also responded positively to the filtering process, exhibiting similar performance with 86.58% accuracy. On the “Original + Filtered” dataset, YOLO11n-cls achieved the highest accuracy rate (87.48%), indicating that data diversity supports the model’s generalization success.

**Figure 9:**
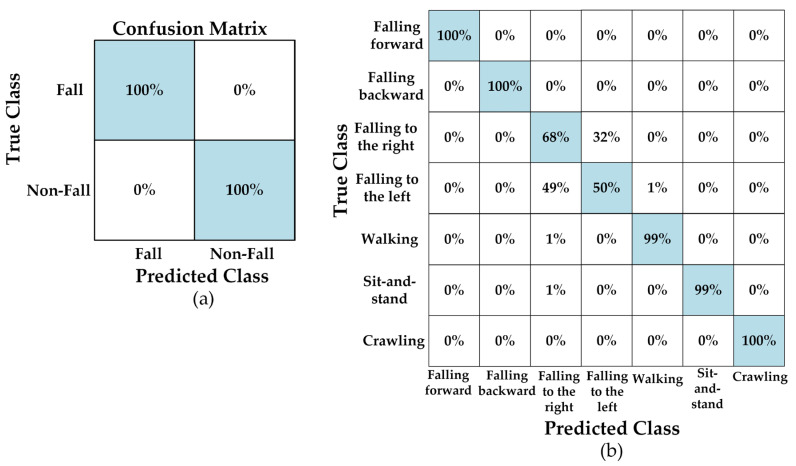
The confusion matrix results of (**a**) binary (YOLO11) and (**b**) multi-class (YOLOv8) classifications for original fall data during testing process.

When examining the binary classification results, it is seen that all models achieved relatively-high-accuracy rates. The YOLOv8n-cls model is the most stable and, generally, the highest performing model, achieving 99.72% accuracy on the original dataset, 96.14% on the filtered dataset, and 98.84% on the combined dataset. The YOLO11n-cls model achieved a near-perfect result with 100% accuracy on the original data, but showed a slight decrease on the filtered and combined datasets (94% and 97.28%). This situation indicates that the class distinction on the original dataset is learned very well by the model, but data diversity and feature distribution after filtering partially reduced this excellence. Figure 9 shows the confusion matrix for each classification result after the testing process.

Figure 9 shows that there are classification mistakes between “falling to the right” and “falling to the left” classes. The model still detects the fall; however, it cannot determine the direction of the fall as in the validation process.

As mentioned above, the limitations of CW radar cause low classification performance between “falling to the right” and “falling to the left” classes. Therefore, a new dataset (six classes) is created to overcome this issue by combining “falling to the right” and “falling to the left” classes into one class (fall to the side). The new dataset had six classes, and the best classification YOLO architecture is used for classification process. According to the results, the YOLOv8n-cls architecture achieved 99.14% and 96.14% classification accuracies on validation and test datasets, respectively. The multi-class classification performance increased to 96.14% from 88.02%. There is an increment of 8.12% classification performance after “falling to the right” and “falling to the left” classes were combined. These results demonstrate that the performance limitation is not caused by the YOLO-based classification architecture itself, but rather by the inherent characteristics of the CW radar signal.

### 4.4. Simple CNN-Based Model

In this section, a simple CNN architecture is designed to classify the original dataset used. The model consists of four convolutional layers. The first convolutional layer uses 16 filters, followed by 32 filters in the second layer, 64 filters in the third layer, and 128 filters in the fourth layer. All convolutional layers employ 3 × 3 kernels with ReLU (Rectified Linear Unit) as the activation function. Each convolutional layer is followed by a 2 × 2 max-pooling layer to progressively reduce the spatial dimensions.

After the convolutional and pooling operations, the extracted feature maps are reduced to a 1 × 1 spatial dimension using an Adaptive Average Pooling layer. This provides a fixed-size feature representation regardless of the input size. The resulting features are then flattened and passed through fully connected layers. The fully connected part of the network includes a dense layer with 128 neurons, followed by a ReLU activation function and a dropout layer with a rate of 0.4 to reduce overfitting. The final layer is a fully connected layer with seven neurons, corresponding to the number of classes, and uses a softmax activation function to produce class probabilities. During training, the cross-entropy loss function (CrossEntropyLoss) is used, and the SGD optimizer is employed with a learning rate of 1 × 10^−2^. Early stopping is applied with a patience of 20 epochs to prevent overfitting, and training is terminated when the validation loss does not improve. The obtained binary and multi-class classification results are presented in Table 8.

Table 8 shows that the performance of the proposed simple CNN architecture on the original dataset is noticeably lower compared to the YOLO-based models, particularly for the multi-class classification task. The average test accuracy of 64.86% indicates that the model has limited capability in distinguishing between multiple classes. In contrast, the binary classification results are significantly higher, achieving an average test accuracy of 94.86%.

The relatively lower performance can be attributed to the simplicity of the CNN architecture, which consists of a limited number of convolutional layers and lacks advanced feature extraction mechanisms. Unlike YOLO-based models, which benefit from deeper architectures, pretrained weights, and more sophisticated feature representations, the proposed CNN is trained from scratch and may not effectively generalize complex patterns on the dataset.

Therefore, these results highlight the importance of using more advanced deep learning architectures for complex classification tasks. The comparison also demonstrates that while simple CNN models can serve as a baseline, state-of-the-art models such as YOLO provide significantly better performance, especially in multi-class scenarios.

### 4.5. LOSO Analysis on the Original Dataset

In the initial experiments, five-fold cross-validation is used to evaluate classification performance. However, in order to evaluate the subject-independent generalization capability of the proposed model, the Leave-One-Subject-Out (LOSO) cross-validation strategy is additionally employed.

The Leave-One-Subject-Out (LOSO) cross-validation procedure is performed over ten iterations. In each iteration, one subject is systematically excluded from the dataset and used as the test set, while the remaining subjects are used for training. Specifically, in the first iteration, the first subject is reserved for testing as the unseen subject, and the rest are used for model training; in the second iteration, the second subject is left out for testing as the unseen subject, and this process continues until each subject is used exactly once as the unseen test set. The results for each subject are presented in Table 9.

According to the LOSO results in Table 9, the average validation and test accuracy for the multi-class classification task are obtained as 92.48% and 85.75%, respectively. For the binary classification task, the average validation accuracy reached 99.56%, while the test accuracy is calculated as 98.54%.

When compared to the five-fold cross-validation results, performance decreases of 2.27% and 1.46% for multi-class and binary classifications are observed under the LOSO setting. This decline is expected, as LOSO represents a more challenging and realistic evaluation scenario by preventing subject-specific data leakage and enforcing strict subject independence. Despite this, the proposed model maintains a high level of performance, demonstrating its robustness and generalization capability across unseen subjects.

### 4.6. Glasgow Dataset Experiments

In this section, classification is performed using the YOLOv8n-cls and YOLO11n-cls models on the “Radar signatures of human activities” dataset (six classes, 1636 samples) provided by the University of Glasgow, employing a five-fold cross-validation approach. The dataset consists of six different classes: “drink,” “fall,” “pickup,” “sitting,” “standing,” and “walking.” Detailed information regarding the data acquisition process and the experimental setup is presented in [35].

**Figure 10:**
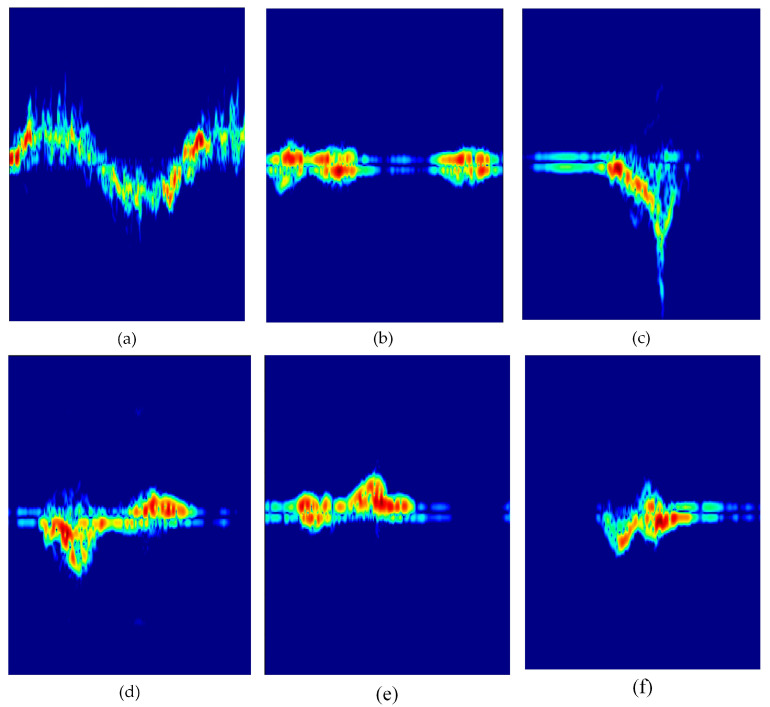
Sample spectrogram images from Glasgow dataset: (**a**) walking, (**b**) drink, (**c**) fall, (**d**) pickup, (**e**) sitting, (**f**) standing.

The spectrogram images were generated from the signals in the dataset. During image generation stage, amplitude extraction and mean removal operations were applied to suppress basic noise and DC components in the signals. In addition, low-frequency components were eliminated using a moving average-based filter. Subsequently, the Short-Time Fourier Transform (STFT) was applied to obtain a time–frequency representation. The resulting spectrograms were expressed on a logarithmic scale, and percentile-based clipping and normalization techniques were applied to reduce the effect of outliers. The spectrogram images in Figure 10 obtained through these processes were then classified. The multi-class classification and binary (fall and others) classification processes were applied to the “Radar signatures of human activities” dataset, and the classification results are presented in Table 10.

Table 10 shows that the YOLOv8n-cls model achieved an average validation accuracy of 93.42% and a test accuracy of 92.18% for the multi-class classification task. The results across the five folds indicate a relatively stable performance, with only minor fluctuations between folds. This consistency suggests that the model generalizes reasonably well despite the complexity of distinguishing between six different human activity classes.

In contrast, for the binary classification task, the YOLO11n-cls model demonstrated significantly higher performance, achieving an average validation accuracy of 99.52% and a test accuracy of 99.16%. The results are consistently high across all folds, with several instances reaching perfect classification (100%). This indicates that the model is highly effective in distinguishing between two classes, likely due to the reduced complexity of the classification problem.

Overall, the results show that the filtering process reduces the risk of overfitting the models and enables the production of more balanced and generalizable results on the test set. The similar accuracy rates of the YOLOv8n and YOLO11n models for multi-class classification reveal that both models have a high level of learning capability. On the other hand, the inferior test accuracies of the YOLOv5n-cls model indicate that the older generation architecture does not have the same generalization power as the newer versions. In conclusion, the findings obtained on the test data show that the YOLOv8n and YOLO11n models provide reliable results with high-accuracy rates for both multi-class and binary classification tasks. The filtering process made a positive contribution, especially in terms of data quality and model stability, helping the system to become more robust against real-world data. The results demonstrate that both models are capable of achieving high accuracy for radar-based human activity recognition tasks.

Table 11 presents a comparison of the results obtained using the proposed method with those from recent studies in the literature.

### 4.7. YOLO Performance on Edge Devices

In this study, the datasets were trained and tested on a computer equipped with an Intel i7-12700 processor and an NVIDIA RTX 3060 graphics card. After the training process, the YOLOv8n-cls model is obtained with a size of 2.915 MB, while the YOLO11n-cls model had a size of 3.122 MB. Each model was evaluated in terms of inference performance on both CPU and GPU within the same computer as well as on embedded platforms such as Jetson Nano. The models for multi-class classification are used during this process. The results obtained for spectrogram image classification provide a comparative analysis of how different hardware platforms influence inference time, as shown in Table 12.

The results demonstrate that inference performance is highly dependent on computational power. While high-end GPUs such as RTX 3060 achieve real-time performance with significant margins, embedded systems like Jetson Nano provide limited but still feasible performance when GPU acceleration is utilized. CPU-only execution on embedded devices is not suitable for real-time applications. Additionally, the performance gap between YOLOv8n-cls and YOLO11n-cls remains minimal, indicating that both architectures are similarly efficient for edge deployment scenarios.

These findings highlight that although the model architecture remains constant, the inference performance is highly dependent on the hardware platform. Consequently, selecting appropriate hardware is crucial, especially for real-time applications where low latency is required.

### 4.8. Limitations and Practical Challenges

This section discusses the limitations of the proposed method and the practical challenges that may arise in real-world deployment. Despite the promising results, several factors can affect the robustness and generalization capability of the model in practical scenarios. Radar placement plays a critical role in the quality of the captured micro-Doppler signatures; slight changes in position or orientation may lead to variations in the observed patterns. Furthermore, the inability of the CW radar to detect localization is another limitation. Additionally, environmental interference and clutter in real home environments can introduce noise, potentially affecting the performance of the model. Another important factor is subject variability, as differences in body size, motion style, and behavior can influence the generated radar signatures.

## 5. Conclusions

The rapid increase in elderly population worldwide has intensified the need for reliable and non-intervention fall detection systems. This study proposes a CW radar-based fall and daily activity recognition framework using micro-Doppler signatures generated from real home environments.

Most previous radar-based fall detection studies relied on CNN, LSTM, hybrid CNN-LSTM architectures or rule-based approaches. This study used YOLO architectures, traditionally designed for image-based object detection, for radar spectrogram classification. To our knowledge, this is the first study to specifically adapt YOLO models for radar-based fall and activity classification. The findings confirm that YOLO-based models are not limited to traditional image detection tasks and can be successfully extended to radar signal representations. This extends the applicability of object detection architectures to time–frequency radar analysis and offers a new perspective for radar-based human activity recognition research.

Future studies will focus on increasing the dataset size, further exploring different YOLO variants for lightweight deployment, and evaluating real-time embedded applications. Additionally, transfer learning and field adaptation techniques can further enhance robustness across unseen house layouts and radar configurations. Consequently, this study highlights the feasibility and effectiveness of YOLO architectures for CW radar-based fall detection and multi-class activity recognition, supported by cross-subject validation and independent dataset verification, providing a new methodological direction to the literature.

## Figures and Tables

**Table 1:** Subjects’ profiles.

Subject	1	2	3	4	5	6	7	8	9	10
Gender	M	F	F	M	M	M	F	M	F	M
Age	38	35	24	36	30	18	29	27	22	32
Height	1.77	1.60	1.69	1.75	1.80	1.80	1.58	1.72	1.64	1.79
Weight	90	58	60	88	81	77	63	76	57	82

**Table 2:** Spectrogram parameters.

Parameters	Value
FFT Points	256
Window Length	256
Overlap Length	192
Window Type	Hamming

**Table 3:** The number of images in each dataset.

	Number of Images					
Dataset	Multi-Class Classification			Binary Classification		
	Training	Validation	Test	Training	Validation	Test
**Original**	490	70	140	490	70	140
**Filtered**	490	70	140	490	70	140
**Original + Filtered**	980	140	280	980	140	280

**Table 4:** The comparison between used YOLO models [34].

Features	Model		
	YOLOv5n	YOLOv8n	YOLO11n
Input size	640 × 640	640 × 640	640 × 640
Backbone	CSP-Darknet	C2f-based backbone	Optimized C2f-based backbone
Anchor usage	Yes	Anchor-free	Anchor-free
Classification Head	Conv + FC	Global AvgPool + FC	Optimized GAP + FC
Parameters (M)	~2.6	~3.2	~2.6
FLOPs (B)	~4.3	~4.6	~4.0

**Table 5:** Classification results obtained with YOLOv8 sub-models for original spectrogram dataset.

Original Spectrogram Dataset	Validation Accuracies for Each Model (%)					Test Accuracies for Each Model (%)				
5-fold cross validation	nano	small	medium	large	xlarge	nano	small	medium	large	xlarge
Fold1	91.4	88.6	92.9	88.6	91.4	85	84.3	82.1	83.6	85
Fold2	95.7	95.7	94.3	94.3	91.4	90.7	84.3	87.1	88.6	84.3
Fold3	91.4	87.1	88.6	90	90	90	87.1	88.6	87.9	87.1
Fold4	92.9	94.3	90	90	91.4	87.9	90	87.9	87.1	86.4
Fold5	92.9	91.4	95.7	94.3	94.3	86.5	90	87.1	90	90.7
Average Accuracy	92.86	91.42	92.3	91.44	91.7	88.02	87.14	86.56	87.44	86.7

**Table 6:** Validation results obtained with YOLO nano sub-models for all datasets.

Data	5-Fold Cross-Validation	Multi-Class Classification			Binary Classification		
		Validation Results (Classification Accuracy) (%)					
		yolov5n-cls	yolov8n-cls	yolo11n-cls	yolov5n-cls	yolov8n-cls	yolo11n-cls
**Original**	Fold 1	87.9	91.4	92.9	100	100	100
	Fold 2	90	95.7	92.9	100	100	100
	Fold 3	90	91.4	88.6	100	100	100
	Fold 4	90	92.9	91.4	100	100	100
	Fold 5	92.8	92.9	94.3	100	98.6	100
	Average Accuracy	90.14	92.86	92.02	100	99.72	100
**Filtered**	Fold 1	86.4	85.7	95.7	97.9	100	100
	Fold 2	92.9	94.3	95.7	96.9	100	100
	Fold 3	95	91.4	90	97.9	100	100
	Fold 4	91.4	87.1	87.1	97.9	100	100
	Fold 5	92.9	91	92.9	98.6	98.6	100
	Average Accuracy	91.72	89.9	92.28	97.84	99.72	100
**Original + Filtered**	Fold 1	88.9	87.9	86.4	99.6	100	100
	Fold 2	91.1	93.6	94.3	100	100	100
	Fold 3	92.9	87.1	84.3	99.6	100	100
	Fold 4	92.1	84.3	87.1	99.3	100	100
	Fold 5	92.9	91.4	90.7	98.6	98.6	99.3
	Average Accuracy	91.58	88.86	88.56	99.42	99.72	99.86

**Table 7:** Test results obtained with YOLO nano sub-models for all datasets.

Data	5-Fold Cross-Validation	Multi-Class Classification			Binary Classification		
		Test Results (Classification Accuracy) (%)					
		yolov5n-cls	yolov8n-cls	yolo11n-cls	yolov5n-cls	yolov8n-cls	yolo11n-cls
**Original**	Fold 1	42.9	85	86.4	54.3	99.3	100
	Fold 2	42.9	90.7	89.3	42.9	100	100
	Fold 3	67.9	90	81.4	66.4	99.3	100
	Fold 4	30	87.9	87.9	89.3	100	100
	Fold 5	31.4	86.5	85.7	86.4	100	100
	Average Accuracy	43.02	88.02	86.14	67.9	99.72	100
**Filtered**	Fold 1	45	86.4	87.9	97.8	96.4	93.6
	Fold 2	82.9	87.9	87.1	71.4	96.4	85.7
	Fold 3	86.4	88.6	85	68.6	97.9	95.7
	Fold 4	14.2	88.6	87.9	42.9	96.4	97.1
	Fold 5	60.7	86.4	85	90	93.6	97.9
	Average Accuracy	57.84	87.58	86.58	74.14	96.14	94
**Original + Filtered**	Fold 1	83.2	83.9	87.5	98.6	98.2	96.4
	Fold 2	78.2	87.9	87.1	100	100	99.6
	Fold 3	79.6	86.8	86.8	96.1	97.1	97.1
	Fold 4	66.4	89.3	84.6	97.5	98.9	95.4
	Fold 5	33.21	87.5	91.4	97.5	100	97.9
	Average Accuracy	68.12	87.08	87.48	97.94	98.84	97.28

**Table 8:** Simple CNN-based model results for original dataset.

Data	5-Fold Cross-Validation	Multi-Class Classification		Binary Classification	
		CNN		CNN	
		Validation Accuracy	Test Accuracy	Validation Accuracy	Test Accuracy
Original	Fold 1	0.634	0.643	0.973	0.943
	Fold 2	0.804	0.686	0.982	0.943
	Fold 3	0.696	0.671	0.991	0.957
	Fold 4	0.616	0.6	0.982	0.957
	Fold 5	0.616	0.643	0.982	0.943
	Average Accuracy	0.6732	0.6486	0.982	0.9486

**Table 9:** Validation and test results obtained with LOSO for original dataset.

Data	LOSO	Multi-Class Classification		Binary Classification	
		Validation Accuracy	Test Accuracy	Validation Accuracy	Test Accuracy
		yolov8n-cls	yolov8n-cls	yolo11n-cls	yolo11n-cls
Original	Subject 1	0.921	0.843	1	0.971
	Subject 2	0.937	0.886	1	0.971
	Subject 3	0.921	0.8	1	1
	Subject 4	0.937	0.771	1	0.985
	Subject 5	0.921	0.886	0.978	0.971
	Subject 6	0.904	0.829	1	1
	Subject 7	0.921	0.886	1	1
	Subject 8	0.889	0.914	1	1
	Subject 9	0.905	0.9	1	0.985
	Subject 10	0.9921	0.86	0.978	0.971
	Average Accuracy	0.92481	0.8575	0.9956	0.9854

**Table 10:** Validation and test results obtained for Glasgow Dataset.

Data	5-Fold Cross-Validation	Multi-Class Classification		Binary Classification	
		Validation Accuracy	Test Accuracy	Validation Accuracy	Test Accuracy
		yolov8n-cls	yolov8n-cls	yolo11n-cls	yolo11n-cls
Original	Fold 1	0.933	0.92	0.988	1
	Fold 2	0.945	0.921	1	0.988
	Fold 3	0.933	0.921	0.994	1
	Fold 4	0.927	0.945	0.994	0.982
	Fold 5	0.933	0.902	1	0.988
	Average Accuracy	0.9342	0.9218	0.9952	0.9916

**Table 11:** Comparison of proposed method with other studies.

Study	Year	Radar	Experimental Environment	Data Type	Data Amount	Subject	Motion	Method	Accuracy (%)
Jokanović and Amin [24]	2017	FMCW	Laboratory	Private dataset	408	3	4	Auto encoder	97.1
Maitre et al. [26]	2021	UWB	Laboratory	Private dataset	660	10	5	CNN-LSTM	90
Kittiyanpunya et al. [27]	2023	FMCW	Home	Private dataset	270	10	9	LSTM	99.5
Shen et al. [28]	2024	FMCW	Laboratory	Private dataset	360,000 Frames	15	3	Rule-based (Dynamic DBSCAN)	96.3
Li et al. [29]	2024	CW	Laboratory	Private dataset	4435	97	7	CNN-BiLSTM	98.83
Cho et al. [30]	2025	CW	Laboratory	Private dataset	2192	5	11	BNN	93.1
This study	2026	CW	Home	Private dataset	700	10	7	Spectrogram-YOLO	^a^ 100/^b^ 88.02

^a^ Fall/non-fall classification; ^b^ multi-class classification.

**Table 12:** Inference time comparison on different platforms.

Parameter/Hardware	Nvidia RTX3060 6GB-GPU		i7-12700 Gen 16GB-CPU		Jetson Nano 4GB-GPU		Jetson Nano 4GB-CPU	
	yolov8n-cls	yolo11n-cls	yolov8n-cls	yolo11n-cls	yolov8n-cls	yolo11n-cls	yolov8n-cls	yolo11n-cls
Model Size (MB)	2.915	3.122	2.915	3.122	2.915	3.122	2.915	3.122
Compute Power (TFLOPS)	~12.7 TFLOPS		~1.52 TFLOPS		~0.5 TFLOPS		~0.01–0.02 TFLOPS	
Input Size (pixel)	640 × 640							
Inference Time (s)	0.00799	0.00951	0.01201	0.016	0.05886	0.05901	0.35721	0.35824

## Data Availability

The raw data supporting the conclusions of this article will be made available by the authors on request.

## Abbreviations

YOLO	You Only Look Once
CNN	Convolutional Neural Networks
FFT	Fast Fourier Transform
FSK	Frequency Step Modulation
IMU	Inertial Measurement Unit
FMCW	Frequency-Modulated Continuous Wave
UWB	Ultra-Wideband
LSTM	Long Short-Term Memory
BNN	Binary Neural Network
LOSO	Leave-One-Subject-Out
